# Sea Conch Peptides Hydrolysate Alleviates DSS-Induced Colitis in Mice through Immune Modulation and Gut Microbiota Restoration

**DOI:** 10.3390/molecules28196849

**Published:** 2023-09-28

**Authors:** Hidayat Ullah, Ting Deng, Muhsin Ali, Nabeel Ahmed Farooqui, Duaa M. Alsholi, Nimra Zafar Siddiqui, Ata Ur Rehman, Sharafat Ali, Muhammad Ilyas, Liang Wang, Yi Xin

**Affiliations:** 1 Department of Biotechnology, College of Basic Medical Science, Dalian Medical University, Dalian 116044, China; hidayat.khan89@yahoo.com (H.U.); dtings123@163.com (T.D.); muhsinaliswati65@gmail.com (M.A.); nabeel.farooqui99@yahoo.com (N.A.F.); doaa.alshouly@gmail.com (D.M.A.); nimra.siddiqui12@gmail.com (N.Z.S.); ata_burraq@yahoo.com (A.U.R.); muhammad.ilyas134h@gmail.com (M.I.); 2 Department of Biochemistry and Molecular Biology, College of Basic Medical Science, Dalian Medical University, Dalian 116044, China; sharafat051@gmail.com; 3 Stem Cell Clinical Research Center, National Joint Engineering Laboratory, Regenerative Medicine Center, The First Affiliated Hospital of Dalian Medical University, Dalian 116011, China

**Keywords:** inflammatory bowel disease, dextran sulfate sodium, peptides, intestinal inflammation, gut microbiota

## Abstract

Inflammatory bowel disease (IBD) is a persistent, lifelong inflammation of the digestive system. Dextran sulfate sodium is commonly used to induce colitis in experimental animal models, which causes epithelial damage, intestinal inflammation, mucin depletion, and dysbiosis of the gut microbiota. Various prebiotics, polysaccharides, and polypeptides are used for IBD treatment. In this study, we used a murine model utilizing BALB/c mice, with 10 mice per group, to investigate the treatment effect of sea conch peptide hydrolysate (CPH) on DSS-induced colitis mice. Colitis was induced through the administration of 2.5% DSS in drinking water over a seven-days period. Furthermore, on the eighth day of the experiment, sea conch peptide hydrolysate (CPH) at low (100 mg/kg), medium (200 mg/kg), and high (400 mg/kg) doses, which were continued for 14 days, were assessed for medicinal purposes in DSS-induced colitis mice. Our results showed that CPH treatment significantly alleviated the severity and symptoms of colitis. The epithelial integrity and histological damage were improved. Intestinal inflammation and inflammatory cell infiltration were improved. Furthermore, the expression of pro-inflammatory cytokines was reduced, and intestinal barrier integrity was restored by elevating the tight junction proteins. Moreover, 16s RNA sequencing revealed dysbiosis of the gut microbiota was observed upon DSS treatment, which was reinstated after CPH treatment. An increased level of *Firmicutes* and *Lactobacillus* was observed in the treatment groups. Finally, our results suggest that CPH would be recommended as a functional food source and also have the potential to be used as a medicinal product for different gastrointestinal disorders.

## 1. Introduction

Inflammatory bowel syndrome (IBS) is a prevalent health problem in Western societies, affecting more than two million Americans. It comprises ulcerative colitis (UC) and Crohn’s disease (CD) and is dependent on various factors, distinguished by gastrointestinal tract inflammation and a compromised immune system [1,2]. The exact mechanism of IBD is unclear and remains up for discussion. However, existing evidence from different studies supports the hypothesis that the host’s acquired immunity interacts intricately with environmental exposure, impaired epithelial barrier function, dysregulated gut microbiota, genetic predisposition, and other factors as probable causes [3,4,5]. Both UC and CD exhibit immunologic, genetic, therapeutic, and phenotypic variations. While Crohn’s disease is characterized by abdominal cramps and bloody mucoid diarrhea [6], the symptoms of ulcerative colitis are limited to rectum and colon inflammation as well as fatigue, diarrhea, and rectal bleeding [7]. Current medical treatment for inflammatory bowel disease includes anti-diarrheal agents, amino-salicylates, anti-inflammatory agents, immunomodulators, biological agents, and antibiotics [6,8,9]. Nevertheless, the current treatment approaches for inflammatory bowel disease (IBD) have negative impacts on the overall health of the host. Prolonged use of these medications can result in immune suppression, increasing the patient’s vulnerability to infections and other illnesses [10,11]. Traditional Chinese medicines, including medicinal mushrooms, have been widely employed for their strong therapeutic effectiveness in treating various human illnesses [12]. Studies also reported the effect of polysaccharides as prebiotics in IBD treatment [13]. Research demonstrated that polysaccharides extracted from the mushroom *Dictyophora indusiata* possess therapeutic properties in countering gut dysbiosis caused by antibiotics. Furthermore, these polysaccharides have improved gut integrity and reduced the inflammatory response, providing beneficial effects [14]. Since 618 A.D., *Dictyophora indusiata* and its bioactive components have been reported to treat numerous diseases. Peptides are responsible for the function and regulation of the different cells in the body. Enzymes then hydrolyze proteins ingested by the human body, and most of the resulting products are absorbed as digested peptides. In 1992, Bayliss and Starling discovered the active peptide secretin in the School of Medicine at the University of London. After their discovery, it was found that peptides have antioxidant, antibacterial, antitumor, and anticoagulant activities. They can also decrease blood pressure and improve immunity [15,16]. 

Research into natural active peptides has mainly been focused on a small number of marines. The progress and improvement in modern molecular drug design and manufacturing have made the discovery of cancer treatment a wonderful goal [17]. A variety of human cancers, such as testicular cancer, pediatric lymphoblastic leukemia, and lymphomas, have already been successfully treated with certain peptides and have seen prolonged survival [18,19]. Although there have been advancements in current therapies with chemotherapy, they are often accompanied by many side effects, motivating the search for alternative, effective treatments with fewer side effects [20]. Natural products represent a rich source of new medicine, chemical entities, and drug leads [21,22]. Approximately 80% of the chemotherapeutic agents and half of all available drugs are based on natural products [23]. A total of 87% of human disorders, including cancer, are treated by natural products [24]. 

Marine sources have significant potential for drug discovery to prevent and treat cancer. After 1980, with the help of biotechnology advancements, the marine source was considered a rich source of new drugs and other applications [25]. Because of this potential, researchers are taking advantage of the complexity and diversity of novel drug research. In general, bioproducts have been the major source of compounds for the treatment of many types of cancer and have also led to the evolution of not only chemical anti-cancer drugs but also a unique and potentially relevant mechanism of action [26,27]. Khan et al. (2022) reported that shrimp peptide hydrolysate (SPH) modulates the cyclophosphamide (CTX) effects on the gut of immunosuppressed mice. SPH increases the immune organ index, enhances the serum level of cytokines (IL6, IL1, TNF-α), IgM, and IgA, reinstates goblet cells and intestinal mucosal integrity, and increases the expression level of tight junction proteins (occludin, ZO-1, claudin-1)and Muc-2. It was also found that SPH treatment shows immune modulation and gut microbiota restoration effects on CTX-induced immune-suppressed mice. 

Oysters are abundant in vitamins, minerals, and proteins. In many areas, it serves as a significant source of high-quality nutrients. Previously reported hydrophobic peptide fractions were isolated from oyster soft tissue by an enzymatic process. Peptides were reported to have no cytotoxicity effect and the best anti-oxidant effect [28]. The veined sea conch, scientifically known as *Rapana venosa*, is a carnivorous sea snail that was originally an invasive species in the Black Sea. However, it has now gained significance as a valuable seafood with considerable nutritional and economic benefits. The primary food source for *Rapana* is the black muscle [29]. Research indicates that the consumption of *Rapana venosa* positively impacts the lipid profile and antioxidant capacities in the serum of rate that are fed an atherogenic diet [30]. A glycosylated functional unit derived from *Hemocyanin venosa* has exhibited an inhibitory effect on the replication of both the epstein-bar virus and the herpes simplex virus (HSV-1) [31]. *Rapana* hemocyanin has been shown to possess more potent antiviral activity when compared to hemocyanin from various other marine species.

Additionally, from *Rapana venosa* hemolymph, four proline-rich peptides have been successfully isolated [32]. Indeed, in addition to its value as seafood and antiviral properties, *Rapana venosa*, or sea conch, is also used in traditional Chinese medicine. The fresh meat of sea conch is employed to treat conditions related to hepatic heat and red eyes, as well as ophthalmalgia, chest pain, and abdomen pain [33]. Its traditional medicinal uses highlight its significance in various aspects of human health and well-being. 

However, to the best of our current understanding, the activity of sea conch peptide hydrolysate on ulcerative colitis is unknown. Thus, this study hypothesizes that the sea conch peptide hydrolysate can help in colitis treatment by modulating the immune response and restoring the gut microbiota. Our study was aimed at exploring the potential of sea conch peptide hydrolysate in colitis treatment by modulating the immune response and restoring the gut microbiota. 

## 2. Materials and Methods

The sea conchs were obtained from the seafood market (in Lvshunkou, Liaoning, and Dalian, China). Trypsin was bought from RHAWN Chemicals in China. Dextran sodium sulfate was purchased from Yeasen Biotechnology in Shanghai, China. Antibodies were bought from Proteintech (Wuhan, China). Different ELISA kits for IL1-β, IL-17, IL-10, TNF-α (Shanghai Jianglai Industrial Share Ltd., Shanghai, China). triazole reagents (Thermo Fisher Scientific Waltham, MA, USA); all other reagents used in experiments were of analytical grade. 

### 2.1. Preparation of Conch Peptide Hydrolysate (CPH) 

CPH was prepared using an enzyme for hydrolysis following the method described by [34] with a slight modification. Briefly, the shell of the conch was removed, and the meat of the conch was minced and ground in a grinder. After grinding, the whole minced meat was washed for one hour at 95 °C. After washing, the minced residues were sieved with a 150 µm filter and mixed with a double volume of distilled water. 1% (*w*/*w*) of trypsin enzyme was added and incubated for 7 h at 50 °C in a water bath under continuous agitation. After 7 h, the enzyme was deactivated at 100 °C for 20 min. The digested lysate was then centrifuged at 11,000 rpm for 20 min at 4 °C to collect the supernatant, and the Bradford method was used to measure the CPH concentration. The CPH supernatant was used to make conch peptide hydrolysate using a lyophilizer machine to convert the supernatant into powder form.

### 2.2. Molecular Mass Distribution of CPH (Liquid Chromatography-Mass Spectrometry) 

For molecular mass determination, the conch peptide hydrolysate was evaluated using liquid chromatography-mass spectrometry (LC-MS). *m*/*z* 200–2000 was the mass range employed for the precursor ion detection. Data from all experiments were collected for the analysis and identification of peptides and proteins in the hydrolysate. 

### 2.3. Experimental Design and Animal Housing 

A total of 50 specific pathogen-free male BALB/c mice, aged 4–5 weeks old, were acquired from Liaoning Changsheng Biotechnology Co., Ltd. (Liaoning, China) The ethical committee of Dalian Medical University approved the use of mice for the experiments. All mice were freely allowed to adapt themselves in the laboratory environment for one week under germ-free conditions at 22 ± 3 °C and 50 ± 5% (RH) relative humidity with 12–12 h of light/dark cycles; they were provided with distilled water and special food for SPF-grade mice provided by MAO HUA BIOLOGY (Shenyang Mao Hua Biotechnology Co., Ltd., Shenyang, China). 

### 2.4. Colitis Induction and Treatment Protocol

After adaptation to the lab environment for one week, all the animals were categorized into five different groups (n = 10 per group), comprised of normal control (NC), model group (DSS group), CPH low dose (LCP), CPH medium (MCP) dose, and CPH high dose (HCP). Ulcerative colitis was induced using 2.5% dextran sulfate sodium (DSS) with a molecular weight range of (36,000–50,000) in autoclaved distilled water and administered for 7 days. The negative control group was given autoclaved drinking water. On the 8th day, all the DSS water was replaced by distilled water, and all animals in the three groups except the normal and DSS groups were gavaged with 100,200, and 400 mg/kg of conch peptide hydrolysate daily for 15 days. Throughout the treatment period (15 days), the normal control and DSS groups received the same dosage of PBS orally. On day 22, all the animals were sacrificed, the colon swiftly removed, and washed with cold PBS. The distal portion was preserved in 4% formalin for histological examination, while the remaining parts were stored at −80 °C for further assays.

### 2.5. Disease Activity Index Measurement 

The severity and symptoms of colitis were examined daily to assess the disease activity index, as previously described by [35]. Body weight, stool consistency, diarrhea, bloody stool, and rectal bleeding were examined, and the disease activity index reflected the disease, as shown in Table 1.

### 2.6. Body Weight, Organ Indexing, Food, and Water Intake Determination

During the experiment, the mice’s body weight was noted daily. Food and water intakes were measured every three days. After sacrificing the animals by cervical displacement, organ indexing for different organs like the spleen, thymus, colon, and small intestine was calculated using the following formula:

Organ index (mg/g) = weight or organ (mg)/weight of mouse (gm)

### 2.7. Histopathological Examination of the Colon 

After sacrificing the animals, the colon was collected, and 3 µm thick tissue sections were fixed in 4% formalin at room temperature for 24 h. The tissue was then deparaffinized in xylene twice for 10 min and rehydrated through different ethanol gradients. Afterward, the tissue was subjected to hematoxylin and eosin (HE) staining. Following this staining process, histological alterations were observed using a microscope (Leica Microsystems, Wetzlar, Germany). 

### 2.8. Mucin Analysis, Goblet Cells, and Mucus Layer Thickness

To assess Mucin-2 expression in colon tissue, immunohistochemistry (IHC) was performed. The 5 µm paraffin-embedded colon tissue was cut and placed onto positively charged slides. Slides were deparaffinized in xylene and rehydrated in a decreasing gradient of ethanol, following the protocol of the immunohistochemical staining kits SP-KIT9720 (MXB Biotechnologies Biotechnology, Beijing, China), according to manufacturer instructions. A semi-quantitative method was used to analyze the data. Each slide was observed randomly three times in different fields for immunolabeled cells. Periodic acid staining (PAS) was employed to assess the goblet cells and mucous epithelium thickness in the colon tissue. The slides underwent deparaffinization using xylene and were then rehydrated through a series of ethanol gradients. Next, they were treated with periodic acid for five minutes at room temperature. Slides were washed by using ultra-high filter water three times 6 × 3. Schiff reagents were then applied to slides for 10 min at room temperature, and slides were covered in a box, followed by 8 min of washing under running water. Counterstain with hematoxylin was applied, washed under running tap water for 7 min, and then dehydrated with ethanol and transparent treatment with xylene. Neutral balsam cat-G8590 (solar bio) was used to cover the slides. Histological changes were evaluated by an independent researcher in a blinded manner. 

### 2.9. Myeloperoxidase Activity Assay 

To assess the influx of neutrophils into the colonic tissue, the myeloperoxidase level was measured, which is indicative of the presence of macrophages and monocytes [35]. The colon tissue was weighted, cut, and then homogenized in 0.1M PBS (pH 7.4). The homogenates were then centrifuged at 8000 rpm for 20 min at 4 °C, and the resulting supernatant was collected. Subsequently, MPO levels were evaluated using an ELISA kit (Cusabio Technology, Wuhan, China), following manufacturer instructions. 

### 2.10. Measurement of Cytokine Level in the Colon by ELISA 

The concentration level of different cytokines in colon tissue was quantified using ELISA. Colon tissue was homogenized in a PBS solution and centrifuged at 3500 rpm for 20 min at 4 °C, and the supernatant was collected and stored at −80 °C for further analysis. Different cytokines (IL-17, TNF-α, IL-10, and IL-1β) were analyzed through ELISA (Shanghai Jianglai Industrial Share Ltd. Shanghai, China) following manufacturer instructions. 

### 2.11. Determination of Intestinal mRNA

IL-23, IL-4, IL-22, IL-17, IL-10, and TNF-α expression levels were measured. Total RNA from colon tissue was extracted using triazole reagents (Thermo Fisher Scientific Waltham, MA, USA). The total RNA was stored at −80 °C. cDNA was transcribed using HiScript II Q RT Mix (Vazyme biotech., Ltd. Nanjing, China). ChamQ Universal SYBER qPCR master mix kit was used to run quantitative PCR in the Bioer Light gene 9600 analyzer by Hitech (Beijing, Hangzhou, 310053, China). All primers used in this study were synthesized through Bioengineering Shanghai Co., Ltd. Shanghai, China and shown in Table 2. For normalization, the reference gene β-actin was employed. Each sample was examined three times, and relative expression was calculated and examined using the instrument software gene 9660, as well as group differences, using GraphPad Prism 9.5. 

### 2.12. Immunofluorescent Staining for Tight Junction Protein 

Immunofluorescent staining was performed to evaluate the expression of tight junction proteins, including claudin-1, Zonula Occludin (ZO1), and Occludin. A total of 5 µm paraffin-embedded colon tissue was cut and placed on a positive charge slide. Then kept in xylene for 10 min twice to deparaffinized and rehydrate in a series of gradient-decreasing concentrations of ethanol. Slides were treated in citrate buffer for antigen retrieval in a microwave oven at 350 watts for 15 min. Blocking reagents were used to block the tissue sections of slides for 30 min. After blocking, slides were washed with PBS three times for 5 min and incubated overnight with primary antibodies against ZO1, Occludin, and Claudin (proteintech, Wuhan, China) at 4 °C. After washing, the slides were then incubated with fluorescein (FITC)-conjugated Affinipure goat anti-rabbit (proteintech) secondary antibodies for one hour, and DAPI was used for 5 min to stain the nucleus. Finlay, the slides were examined under a microscope, and images were captured. 

### 2.13. Stool DNA Extraction and 16s RNA Pyrosequencing

Before sacrificing the mice, stool samples from all mice were collected in each group. Total genomic DNA samples were extracted using fresh fecal samples using a FOREGENE stool DNA extraction kit (CAT.NO.DE-05713), following the manufacturer’s instructions. After extraction, DNA was quantified using a nanodrop (Thermo Fisher Scientific, Wantham, MA, USA) and run on a 1% agarose gel to check sample quality. To observe the diversity and composition of the bacterial community in the gut, the V3-V4 region of the 16s RNA gene was targeted for PCR amplification using forward primer 341f (CCTACGGGAGGCAGCAG) and reverse primer 518r (ATTACGCGGCTGCTGG), and sequenced with the Illumina NovaSeq6000 platform at GUHE Info Technology Co., Ltd. (Hangzhou, China). QIIME software was used to analyze the diversity and abundance of different bacterial communities in samples as alpha diversity, including Shannon and richness indexes. In contrast, beta diversity was visually represented by a weighted uniFrac distance based on principal coordinate analysis (PCoA) and non-metric multidimensional scaling (NMDS). Moreover, to determine the key biomarker of each group, LEfSe linear discrimination analysis effect size (LDA) evaluates the abundance and difference. KEGG pathways, FAPROTAX, and bug bases were used to analyze ecologically related metabolites and their functions in prokaryotic clades. 

### 2.14. Statistical Analysis

All data results were presented as the mean ± standard error of the mean. Statistical data analysis was performed using GraphPad Prism version (9.5). A one-way analysis of variance was used to determine significance, followed by Tukey’s multiple comparison test. For 16sRNA data, QIME and R programs were utilized. Additionally, different prokaryotic clades, ecologically relevant functions, and high-level phenotypes were analyzed using STAMP, FAPROTOAX, and Bugbase tools for the statistical analysis of metagenomic profiles. 

## 3. Results 

### 3.1. Molecular Mass Distribution of Conch Peptides Hydrolysate

**Figure 1:**
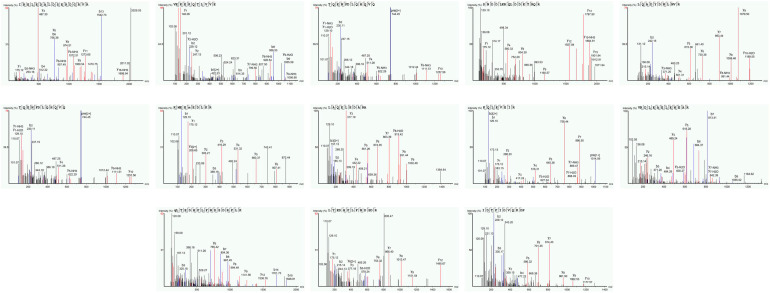
Mass spectrometry analysis for conch bioactive peptides in Hydrolysate. The obtained Peaks corresponded to peptides samples with varying concentrations of different amino acids having distinct molecular mass.

To analyze the conch peptide hydrolysate after digestion with the trypsin enzyme. The conch peptide hydrolysate was freeze-dried and used for mass spectrometry analysis. The mass spectrometry results reveal 13 peaks corresponding to different peptides with varying molecular weights and unique combinations of amino acids, as shown in Figure 1 and Table 3. 

### 3.2. Conch Peptides Hydrolysate Ameliorate Clinical Symptoms in DSS Induce Colitis Mice

**Figure 2:**
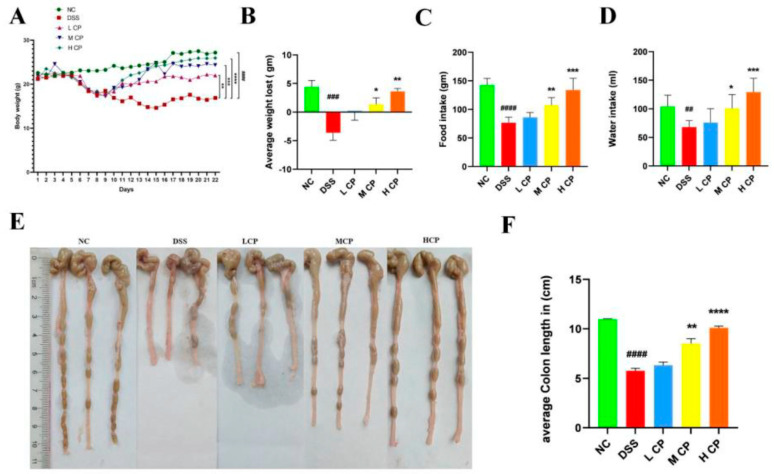
Conch peptides hydrolysate alleviate symptoms of Colitis. (**A**) Body weight (**B**) average weight lost (**C**) food Intake (**D**) water intake (**E**) colon length (**F**) average colon length. Results reflected as mean ± SEM., ## *p* < 0.01, ### *p* < 0.001, #### *p* < 0.0001 comparison to normal. * *p* < 0.05, ** *p* < 0.01, *** *p* < 0.001, **** *p* < 0.0001 comparison to DSS-treated group.

After administering DSS through drinking water, all the mice were examined, and the depicted change in body weight was recorded on a daily basis (Figure 2A,B). The DSS (positive control) group exhibited a significant reduction in body weight (*p* < 0.0001) compared to the normal control. However, treatment with CPH demonstrated a positive effect on body weight, showing improvements in a dose-dependent manner (Figure 2A). In comparison to the DSS group, the LCP, MCP, and HCP groups showed significant improvements in body weight (*p* < 0.01), (*p* < 0.001), and (*p* < 0.0001), respectively. The DSS group experienced weight loss due to severe diarrhea and rectal bleeding compared to the normal control group (*p* < 0.001). However, supplementation with CPH at medium and high doses mitigated the body weight loss, as shown in Figure 2B. DSS intake also affected food and water intake in the positive control (DSS) group, as depicted in (Figure 2C,D). Conversely, CPH treatment led to an increase in food and water intake. Furthermore, the DSS group exhibited a shrinkage in colon length compared to the normal control group (*p* < 0.0001). However, treatment with medium and high doses of CPH resulted in a significant increase in colon length (*p* < 0.01) and (*p* < 0.0001), while low doses showed no significant increase in colon length (Figure 2E,F).

**Figure 3:**
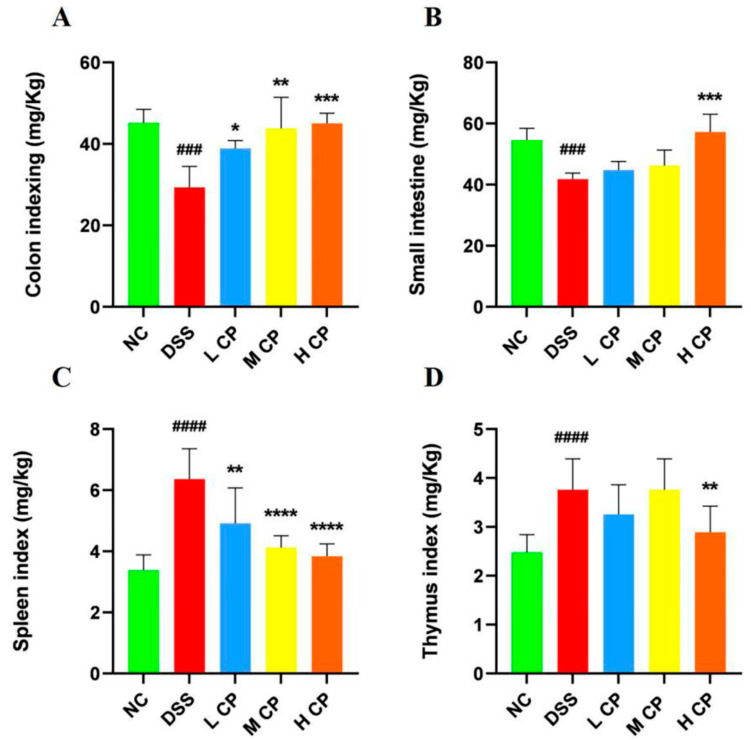
CPH treatment resulted in improved organ indexing in DSS-treated mice. (**A**) Colon index (**B**) small intestine Index (**C**) Spleen Index (**D**) thymus index. Data presented as comparison to normal control, ### *p* < 0.001, #### *p* < 0.0001. while * *p* < 0.05, ** *p* < 0.01, *** *p* < 0.001, and **** *p* < 0.0001, vs. DSS group.

The colon and small intestine indices of the normal control group were notably higher than those of the DSS group (*p* < 0.001). However, treatment with high-dose CPH significantly increased both colon and small intestine indices, *p* < 0.001 (Figure 3A,B). The intake of DSS causes inflammation, leading to an increase in the size of the spleen and thymus and higher indices compared to a normal control. In contrast, CPH treatment showed similar indices to the normal control group (Figure 3C,D).

### 3.3. CPH Improves Histological Changes, Goblet Cell and Mucin Production

**Figure 4:**
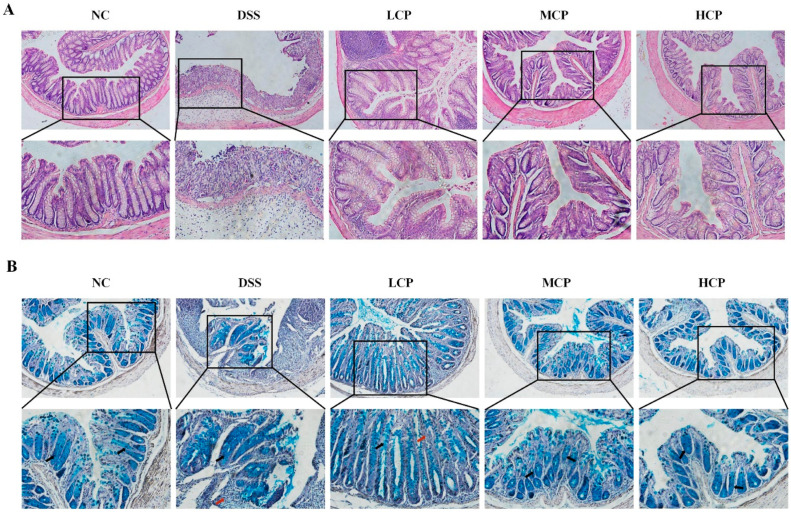
CPH treatment ameliorates histopathological changes by improving TJs expression and goblet cell production in DSS-induced colitis mice. (**A**) H&E images showing the healing effect of CPH on DSS-induced colitis mice magnification (upper 10× (lower 20×). (**B**) shows the Alcian blue staining of colon tissue. The number of goblet cells indicated by the black arrow and inflammatory cell infiltrate indicated by the red arrow was determined in all groups (upper 10×) (lower 20×) magnification.

In order to examine the elevation effect of CPH on mucin expression and goblet cell production in the colon, H&E staining and Alcian blue staining (AB) were performed. The control group displayed a well-defined, tightly packed columnar epithelium with a clear separation between the mucosa and submucosa layers. The presence of deep and narrow shapes, along with a significant number of goblet cells, indicated a normal and healthy histological structure. On the other hand, the DSS-treated group showed significant and severe histological damage, with distorted and shallow crypt structure, depletion of goblet cells, irregular surface epithelium, and inflammatory cell infiltration. There was a larger space between the mucosa and submucosa layers compared to the normal control group. Nonetheless, the CPH treatment exhibited a therapeutic effect, improving the histological damage of the colon and mitigating inflammatory symptoms. The columnar epithelium damage improved in a dose-dependent manner, and there was a decrease in inflammatory cell infiltrations while goblet cell production increased (Figure 4A,B).

**Figure 5:**
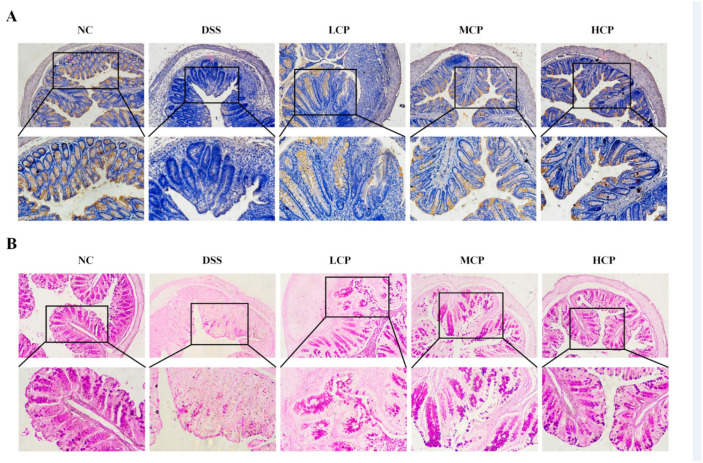
CPH treatment increases mucin expression and replenishes goblet cell production in DSS-induced colitis mice. (**A**) Immunohistochemistry staining for Mucin-2 expression in the colon of different groups. Mucin expression demonstrated by goldish color. (**B**) Periodic acid staining represents the image of colon sections. The number of goblet cells produced was determined in all groups. Magnification (upper 10×) (lower 20×).

Furthermore, immunohistochemistry (IHC) and periodic acid staining (PAS) were performed to assess Mucin-2 expression, neutral mucin, and goblet cell production in the colon. DSS intake caused a severe depletion of Mucin-2 expression. In contrast, the control group revealed a higher expression of Mucin-2 in IHC. However, in the DSS group, the mucin layer and goblet cell production were diminished compared to the normal control. Conversely, the CPH treatment improved the integrity of the intestinal barrier and increased the expression of Mucin-2 and goblet cell production. The DSS group showed a significantly lower number of goblet cells compared to the normal control. At the same time, the effect of CPH treatment, as revealed by PAS staining, demonstrated a significant decrease in glycoprotein content in the DSS group. Nevertheless, the CPH treatment effectively restored and enhanced the production of goblet cells and mucin expression in LCP, MCP, and HCP in a dose-dependent manner, as shown in (Figure 5A,B). 

### 3.4. CPH Regulate Leaky Gut Barrier and Intestinal Inflammation 

**Figure 6:**
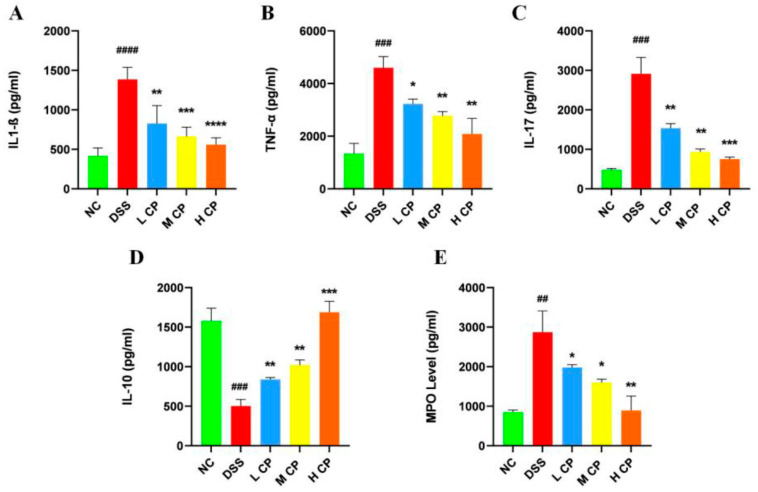
CPH effect on intestinal inflammation and neutrophil infiltration in the colon of DSS-treated mice. Pro-inflammatory cytokines (**A**) IL1-β. (**B**) TNF-α. (**C**) IL-17, and anti-inflammatory cytokines (**D**) IL-10. (**E**) MPO activity levels in colonic mucosa were assessed by ELISA kit.; ## *p* < 0.01, ### *p* < 0.001, #### *p* < 0.0001 vs. normal; * *p* < 0.05, ** *p* < 0.01, *** *p* < 0.001, **** *p* < 0.0001 vs. DSS group. Data presented as mean ± SEM.

To explore the treatment effect of Conch peptide hydrolysate on DSS-induced inflammation, MPO activity, pro-inflammatory cytokines Il-17, TNFα, IL1-β, and the anti-inflammatory cytokine IL-10 were measured in colon tissue using ELISA. MPO activity indicates neutrophil infiltration, and normally, myeloperoxidase is present in lower quantities in monocytes. Figure 6E revealed significantly higher MPO levels (*p* < 0.01) in DSS-treated mice compared to the normal control group. Interestingly, treatment with CPH resulted in a significant reduction in MPO level in a dose-dependent manner (*p* < 0.05, *p* < 0.05, *p* < 0.01) compared to the DSS group. Colitis is characterized by a cascade of chronic inflammation and has been linked to the increased production of pro-inflammatory cytokines, including IL-17, TNF-α, IL-6, IFN-ɤ, and IL1-β, while anti-inflammatory cytokines IL-10, TGF-β, and IL-4 are down-regulated [36,37]. To investigate the treatment effect of CPH on intestinal inflammation, an ELISA assay was performed. The results revealed that the production of pro-inflammatory cytokines was increased, while anti-inflammatory cytokines were decreased in the DSS group compared to the normal control. However, supplementation with CPH at a particularly high dose significantly reduced the production of the pro-inflammatory cytokines IL1-β (*p* < 0.01, *p* < 0.001, *p* < 0.0001), TNF-α (*p* < 0.05, *p* < 0.01), and IL-17 (*p* < 0.01, *p* < 0.001) shown in (Figure 6A–C) and increased the production of the anti-inflammatory cytokine IL-10 (*p* < 0.01, *p* < 0.001), as shown in Figure 6D.

### 3.5. CPH Treatment Regulates mRNA Expression in Colon

**Figure 7:**
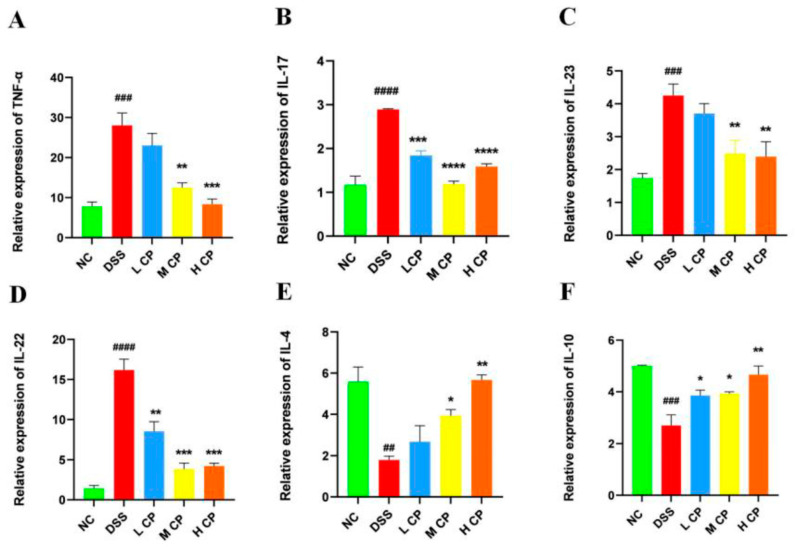
Relative expression of mRNA in colon tissue. (**A**) TNF-α, (**B**) IL-17, (**C**) Il-23, (**D**) IL-22, (**E**) IL4, (**F**) IL-10. mRNA levels were standardized with β-actin expression, and results shown as mean ± SD * *p* < 0.05, ** *p* < 0.01, *** *p* < 0.001, **** *p* < 0.0001 vs. DSS group, ## *p* < 0.01, ### *p* < 0.001, #### *p* < 0.0001 compared with control group.

To further confirm the results, the mRNA expression levels of different cytokines (pro and anti-inflammatory) in the colon were analyzed. The finding revealed higher relative expression levels of pro-inflammatory cytokines, i.e., TNF-α (*p* < 0.001), IL-17 (*p* < 0.0001), IL-23 (*p* < 0.001), and IL-22 (*p* < 0.0001), as shown in Figure 7A–D, respectively, in the DSS group compared to the normal control group. Additionally, the expression level of mRNA for anti-inflammatory cytokines IL-4 *p* < 0.01 and IL-10 *p* < 0.001 was significantly reduced in the DSS group. (Figure 7E,F). In contrast, the CPH treatment groups, especially the high dose, exhibited a significant reduction in mRNA expression for TNF-α (*p* < 0.001), IL-17 (*p* < 0.0001), IL-23 (*p* < 0.001), and IL-22 (*p* < 0.001), while also showing increased mRNA expression for anti-inflammatory cytokines IL-4 (*p* < 0.01 and IL-10 (*p* < 0.01) compared to the DSS group (Figure 7). 

### 3.6. CPH Enhance the Expression of Tight Junction Proteins 

**Figure 8:**
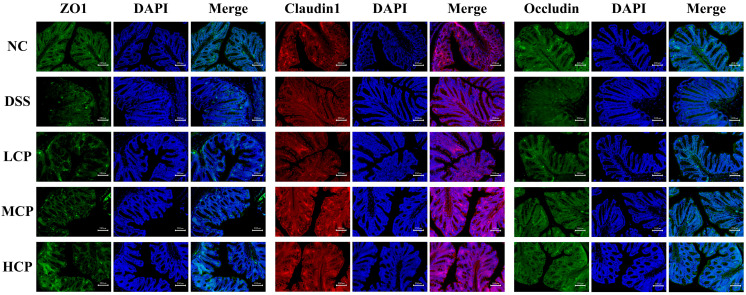
Immunofluorescent analysis of tight junction proteins in the colon tissue of mice. Expression of ZO-1, Claudin1, and Occludin. Pictures magnification 20×, scale bar 200 µm.

Immunofluorescent staining was employed to check the expression of ZO-1, Occludin, and Claudin in colon tissue to check colon permeability. The results revealed a significant decrease in the expression of ZO-1, Occludin, and claudin in the colon of DSS-treated mice. The staining results indicated gut barrier dysfunction (Figure 8). The tight junction protein expression intensity in the DSS group was notably lower than in the normal control. In contrast, after 15 days of CPH treatment, ZO-1, Occludin, and Claudin 1 expression showed significant improvement and restoration in a dose-dependent manner with CPH supplementation, as shown in Figure 8. 

### 3.7. CPH Reinstate Gut Microbiota Dysbiosis 

**Figure 9:**
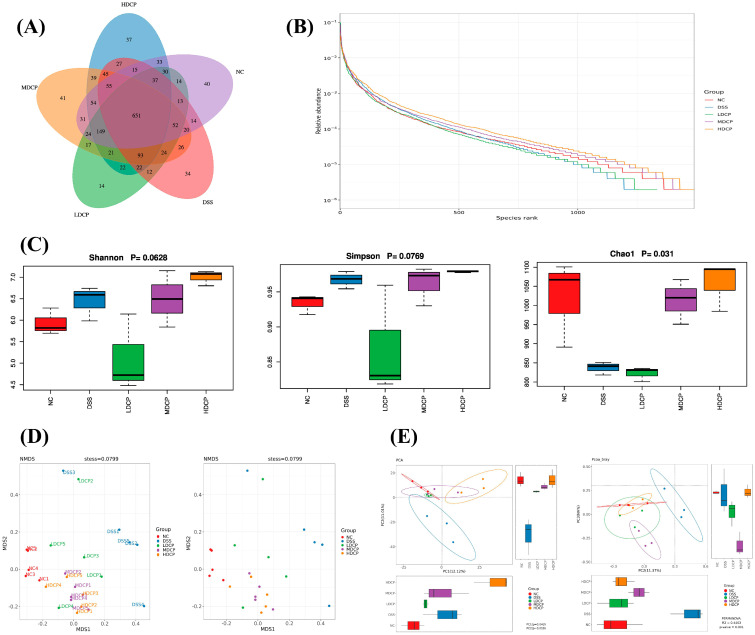
DSS induce Colitis cause dysbiosis by decreasing bacterial community diversity and richness. (**A**) Venn diagram shows the bacteria OTUs shared by different treatment groups. (**B**) Rank abundance curve, show richness and abundance. (**C**) Alpha diversity indices Shannon, Simpson, and Chao to measure richness similarities and dissimilarities. (**D**,**E**) Represent beta diversity index NMDS, PCA, and PCoA plot. Each point shows an individual sample and is differentiated by distinct colors belonging to different groups.

The 16s RNA gene was sequenced using Illumina NovaSeq6000 to study the restorative effect of conch peptide hydrolysate on gut microbiota. In order to characterize the overall structure, changes, patterns, abundance, and richness of different bacterial communities in both the DSS and all CPH treatment groups, the Venn diagram results showed that a total of 1158 OTUs were shared among the normal control and experimental groups. Moreover, variations among the groups were observed. The normal control, LCP, MCP, and HCP groups exhibited a significant elevation in OTUs, while the DSS group showed a decrease in OTUs, as shown in Figure 9A. 

The alpha diversity parameters were evaluated using the rank abundance curve, Shannon, and observed species. Rank abundance and refraction curves were used to assess the bacterial diversity and community richness between experimental groups and controls. In Figure 9B, each treatment group and control group exhibited a unique rank abundance curve. The curve width and horizontal direction orientation indicated the respective species richness and abundance in a distinct manner. Normal control shows high species richness, followed by all CPH treatment groups as compared to DSS groups. Alpha diversity reflects the variation and richness of the gut bacteria. Overall, these findings revealed that CPH treatment improved and enhanced the alpha diversity indices compared to the DSS group. 

Furthermore, beta diversity parameters were determined using non-metric multidimensional scaling (NMDS) and principal component analysis (PCA) to demonstrate the gut microbiota structure and check the inter-sample similarity and dissimilarity of the species composition. Our findings revealed that all the DSS samples were clustered far from the control group. On the other hand, all dose treatment groups in CPH, particularly medium and high dose, were found to be more closely clustered than the normal control group (Figure 9C–E). Beta diversity result analysis reveals that DSS induces colitis and causes dysbiosis, while CPH can help restore microbiota dysbiosis.

**Figure 10:**
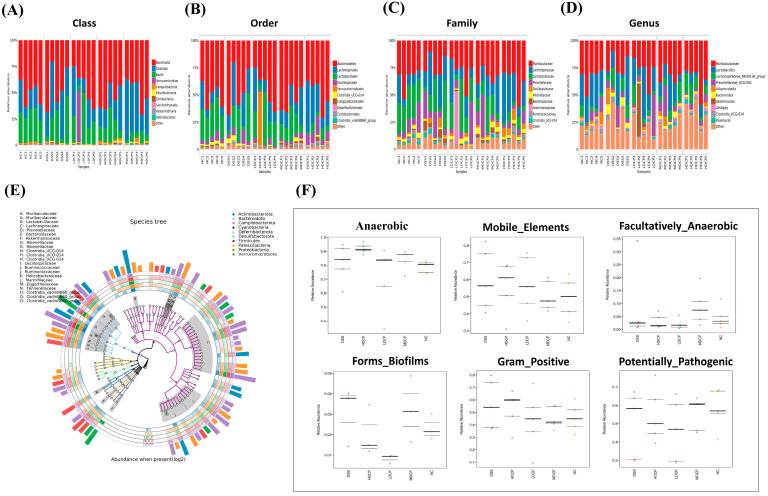
Microbial diversity at different taxonomic levels and Bug bas analysis for different phenotypes. Bacterial count at different taxonomic levels (**A**) class, (**B**) order, (**C**) family, (**D**) genus, (**E**) species tree, (**F**) bug base analysis for different phenotypes.

Moreover, the taxonomic level of bacteria was further observed at the class, order, family, and genus levels (Figure 10A). Results reveal that, compared to the normal control group, the DSS group found an increased abundance of class *Bacterioidia* while decreasing the abundance of class *Bacilli* and class *Clostridia*. Though CPH supplementation reinstates the dysbiosis effectively at a medium and high dose as compared to the DSS group, it increases the abundance of class *Bacilli* and *Clostridia*. Furthermore, the DSS group was observed at the order level for *Bacteroidales* as the dominant order and had a lower abundance of *Lachnocspirales* and *Lactobaclliales* compared to the normal control. In contrast, the treatment groups were found to have the *Lachnocspirales* most dominant bacterial community (Figure 10B). Results exhibited at the family level show the changes among all experimental groups: *Lactobacillaceae*, *Lachnospiraceae*, and *Prevotellaceae* were less abundant in the DSS-treated group as compared to the normal control and treatment groups (Figure 10C).

Interestingly, at the genus level, the DSS-treated group shows a decline in the abundance of *Lactobacillus*, *Lachnospiraceae NK4A136*, and *Prevotellaceae UCG-001* and an increased abundance of *Bacteroides*. In contrast, other treatment groups restored all the changes, as shown in Figure 10D. For further demonstration, a species tree was constructed to show the species richness from the inner circle to the outer circle among different groups, as shown in Figure 10E. To evaluate high-level phenotypes in all experimental groups, bug base analysis identified phenotype differences associated with anaerobic, Gram-positive, mobile elements, facultative anaerobes, biofilm formation, and potentially pathogenic bacteria (Figure 10F). Results reveal that microbiome phenotypes between the normal control group and CPH treatment groups show more or less similarities in the relative abundance of phenotypes. At the same time, DSS-treated groups show high-level variations. The functional genes encoded for containing mobile elements, Gram-positive bacteria, biofilm formation, and anaerobic bacteria were found to be more abundant in the DSS-treated group.

**Figure 11:**
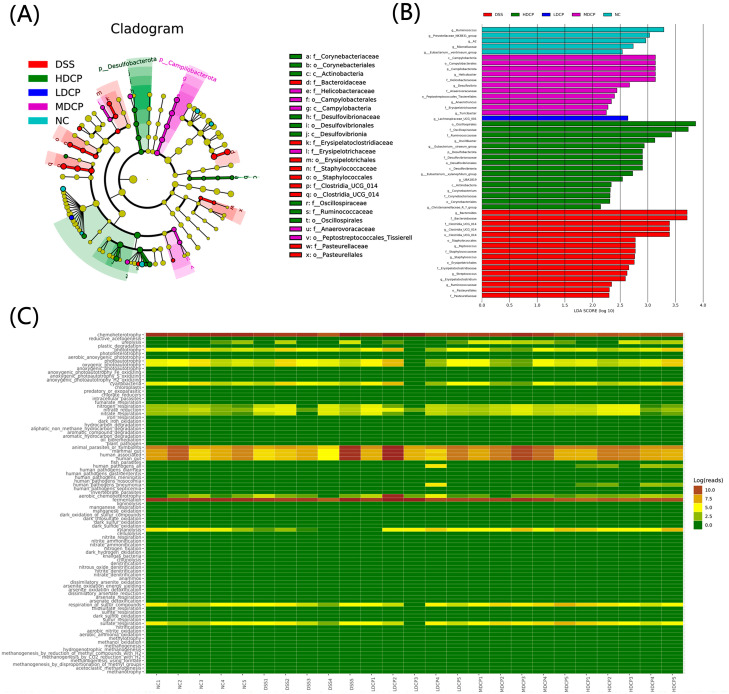
CPH’s modulating effect on gut microbiota. (**A**) Cladogram demonstrating taxa richness among different groups. (**B**) LEfSe analysis bar represents the abundant taxonomic biomarker at different taxa levels. (**C**) Heatmap analysis representing metabolic function and other significant activities in groups.

Furthermore, the taxonomic biomarker was determined by using discriminative analysis effect size. Results reveal that potentially pathogenic *Proteobacteria*, the family *Bacteriodeaceae*, and the genus *Bacteroides* were the most abundant biomarkers in the DSS-treated group. While the normal control groups show the *Ruminococcus* and *Prevotellaceae* as dominant biomarkers, interestingly, the high-dose CPH group shows the *Oscillospirales*, the dominant biomarker, followed by the beneficial bacterial family *Ruminocococcaceae* (Figure 11B). The cladogram tree was constructed to demonstrate the hierarchical relationship of intestinal flora among different groups at different taxonomic levels (Figure 11A). The yellow nodes indicate significant variations across groups of classification units, and the node size reflects the taxon’s relative abundance.

Additionally, FAPROTAX was utilized to predict the metabolic function of the different bacterial communities using 16sRNA sequence results. KEGG pathways show differences between experimental groups. Interestingly, the most enriched pathways were nitrogen respiration, fermentation, sulfur respiration, respiration of sulfur compounds, xylanolysis, and amino-acid biosynthesis. Energy production was altered in various groups, suggesting that CPH treatment can help improve immunity by modulating intestinal microbiota metabolism (Figure 11C). 

## 4. Discussion

Inflammatory bowel disease describes an illness related to ulceration of the mucosal and submucosal layers. One of the most common types of inflammatory bowel disease is ulcerative colitis, which demonstrates superficial mucosal inflammation in the colon, starting in the rectum and moving toward the proximal colon as the disease becomes more severe [5]. DSS is considered to be the most effective way to develop an experimental colitis model. DSS-induced models have been extensively used to study the molecular mechanisms of IBD and search for appropriate treatments. Various natural products, sourced from plants and mushrooms, have been utilized as alternative medicine for the treatment of inflammatory bowel disease, chronic gastritis, ulcerative colitis, and stomach ulcers [38]. Recently, it has been suggested that food may also contain peptides that exhibit biological activity. These food-derived peptides have different beneficial biological activities for human health, such as antihypertensive, antithrombotic, immunomodulatory, and antioxidant activities [39,40]. Therefore, in the current study, the therapeutic effect of sea conch peptide hydrolysate was examined by evaluating gut barrier integrity, immune modulation, and gut microbiota restoration. Trypsin was found to be the most effective enzyme for hydrolysis. Subsequently, the conch peptide hydrolysate was characterized by LC-mass spectrometry, revealing different peptides with varying molecular weights and amino-acid compositions. In accordance with our results, a previously published study identified the different peptide fractions from the hydrolysate by MALDI-TOF analysis [41]. 

In this study, the severity of the disease was demonstrated when 2.5% DSS was administered in the drinking water for 7 days, resulting in the induction of ulcerative colitis. Compared to the normal control group, the DSS-treated group exhibited a significant decrease in body weight, rectal bleeding, diarrhea, and stool consistency. Additionally, a notable shrinkage of the colon was observed. However, supplementation with conch peptide hydrolysate (CPH) showed an ameliorative effect by alleviating the clinical symptoms. The CPH treatment led to an increase in body weight, an increase in colon length, and the healing of other symptoms. Similar results were reported by [35], showing the effect of polysaccharides (DIP) on DSS-induced colitis. 

The spleen and thymus are the major components of the immune system, playing a significant role in nonspecific immunity [42]. Upon activation, differentiation, and proliferation, the weight of the immune organs increases [43,44]. After the intake of 2.5% DSS in drinking water for 7 days, the immune organs (spleen and thymus) were found to increase in size, indicating immune system activation, while the colon and small intestine indices decreased in the DSS group compared to the normal control group. However, after CPH treatment, the immune organ indices decreased, showing the anti-inflammatory response of the peptides, while the colon and small intestine indices increased. Additionally, we observed reduced food and water intake in the DSS group, while the CPH treatment led to an improvement in food and water intake. Our results are in agreement with a previously published study that demonstrated the oyster peptides inhibitory effect on colitis [45]. 

DSS intake causes histological changes, a reduction in the expression of tight junction proteins, increased epithelial apoptosis, and leaky gut symptoms [46,47]. Subsequently, more severe symptoms emerge, such as increased intestinal permeability, excessive bleeding, and a high mortality rate [46,48]. Furthermore, DSS intake also results in goblet cell and mucin depletion, along with the infiltration of granulocytes into the mucosa and submucosa layers. 

In our findings, we examined the histopathological changes: a decline in the production of goblet cells, epithelial layer apoptosis, neutrophil infiltration (MPO activity), and lower TJ expression after DSS intake. Nonetheless, the CPH treatment showed a therapeutic effect and improved the architecture of the colonic tissue, with a noticeable increase in goblet cell production. CPH treatment healed the pathological changes induced by DSS, such as crypt disruption and loss, inflammatory cell infiltration, and mucosal and submucosal edema. A previously published study by [35] supported our results, demonstrating the attenuation effect of polysaccharides on DSS-induced colitis. They observed improvements in chronological changes, intestinal permeability, elevated goblet cells and mucin production, and decreased inflammatory cell infiltration. 

Pro-inflammatory cytokines play an important role in the pathogenesis and development of inflammatory bowel disease (IBD). Numerous studies have reported the pivotal role of these cytokines in driving the chronic inflammation and immune dysregulation observed in IBD [49].

Colitis has been linked to increased expression of several cytokines and chemokines. Previous reports have identified inflammatory cytokines such as IL-17, TNF-α, IL-23, and IL-6 as key players in the development of colitis [50,51].

In the current work, we measured the expression level of different cytokines, including pro-inflammatory and anti-inflammatory ones. Results revealed elevated expression of pro-inflammatory cytokines and an alleviated expression level of anti-inflammatory cytokines. Nevertheless, CPH supplementation markedly reduced the expression of pro-inflammatory cytokines and significantly increased the level of anti-inflammatory cytokines. These results are in agreement with a study published by [13]. Post-treatment with *H. erinaceus* extract shows a therapeutic effect by reducing the expression of inflammatory cytokines and elevating the level of anti-inflammatory cytokines. 

The intestinal epithelium’s role is to simultaneously protect the host from the environment while allowing selective permeability that restricts the movements of harmful molecules but allows the proper absorption of nutrients and water [52]. Tight junctions are protein complexes (Occludin, ZO-1, and Claudin); these complexes act as physiologically active barriers that can change permeability depending on the cellular environment [53,54]. Intestinal barrier dysfunction causes intestinal permeability, which is linked to the pathogenesis of inflammatory bowel disease. Our study reveals similar results: the DSS-treated group shows gut barrier and mucosal layer disruption and alleviation in tight junction protein expression. CPH restores mucosal damage and intestinal barrier integrity with the increased expression of tight junction proteins. 

Gut microbiota plays an important role in the host immune system; physiological activities in the gut and tolerance are all linked with gut microbiota. This has argued for and opened a new platform for characterizing and identifying the intestinal microbiota and their axis with different diseases [5,55,56]. To explore the composition of the gut microbiota, 16sRNA from Illumina NovaSeq6000 was used. Previously published work investigated the richness and diversity of bacterial communities in ulcerative colitis patients as well as in animal models [57]. Another study reported a significant decline in aerobic bacteria species such as *Lactobacillus* in fecal samples of IBS patients and also found a high number of *Proteobacteria* with less abundance of *Lachnospiraceae* [58]. In accordance with these previous results, our results reveal similarity: alpha diversity analysis shows that the normal control group has a high abundance and richness, followed by CPH treatment groups as compared to the DSS treated group, and non-metric multidimensional scaling (NMDS) and principal component analysis (PCA) found great variation among groups. Beta diversity results analysis reveals that the DSS group was clustered far from normal control while the CPH treatment group was closer to normal control; its mean DSS-induced colitis causes dysbiosis, while CPH can help restore microbiota dysbiosis. Also found was a high abundance of *Bacteriodeaceae*, genus *Bacteroides*, in the DSS-treated group, while the normal control was found to have the highest abundance with *Ruminococcus* and *Prevotellaceae* as dominant biomarkers. Interestingly, at the genus level, the DSS-treated group showed a decline in the abundance of *Lactobacillus*, *Lachnospiraceae NK4A136*, and *Prevotellaceae UCG-001* and increased the abundance of *Bacteroides*, while other treatment groups restored all the changes. These results are supported by [59], who reports the abnormal gut microbiota from UC and CD patient samples. Observed depletion of commensal bacteria, particularly *Firmicutes* and *Bacteroides*. Bugbase results demonstrate that microbiome phenotypes between the normal control group and CPH treatment groups show more or less similarity in the relative abundance of phenotypes. At the same time, DSS-treated groups show high-level variations. The functional genes encoded for containing mobile elements, Gram-positive bacteria, biofilm formation, and anaerobic bacteria were found to be more abundant in the DSS-treated group. These results are in accordance with previously published work by [35]. 

In addition, we employed a bioinformatics tool to delve into the metabolome’s function through 16sRNA analysis. Dysbiosis significantly impacts the physiological functions of the body as well as its metabolic and functional pathways. In our study, we utilized STAMP analysis to explore the gut microbiome metabolome in mice. Our findings indicated that CPH has the potential to enhance energy utilization methods, carbohydrate metabolism, and nutrient absorption, suggesting its potential as a therapeutic agent. However, CPH, in its current form, is a hydrolysate and not a pure peptide. This could introduce variability in the composition of the bioactive compounds present in CPH. Further research should explore the isolation and characterization of individual peptides within CPH to gain a deeper understanding of their specific mechanisms.

## 5. Conclusions

In conclusion, our study unveils the therapeutic potential of sea conch peptide hydrolysate (CPH) in effectively ameliorating clinical symptoms and modulating intestinal inflammation in DSS-induced colitis mice. CPH demonstrates its beneficial effects by improving parameters such as body weight, colon length, food and water intake, augmenting mucin production, reinstating intestinal integrity and goblet cells, and exhibiting immunomodulatory properties. These effects encompass a reduction in pro-inflammatory cytokines, an elevation of anti-inflammatory cytokines, and an upregulation of tight junction proteins. Importantly, CPH plays a pivotal role in rebalancing gut microbiota dysbiosis, characterized by a decrease in pathogenic and harmful bacteria coupled with an increase in beneficial bacteria. These compelling findings collectively underscore Conch peptide hydrolysate (CPH) as a promising medicinal prebiotic.

## Figures and Tables

**Table 1:** Disease activity index measurement criteria.

*Score*	*Weight Loss %*	*Stool Consistency*	*Rectal Bleeding*
***0***	None	Normal	Negative
***1***	1–5		
***2***	5–10	Loose stool	Positive
***3***	10–20	Diarrhea	Positive
***4***	>20	Diarrhea	Gross bleeding

**Table 2:** List of Primer used to check mRNA gene expression level.

*Gene*	*Forward Primer*	*Reverse Primer*
*TNF-α*	GGTGCCTATGTCTCAGCCTCTT	GCCATAGAACTGATGAGAGGGAG
*IL 23*	GAGCAACTTCACACCTCCCT	TAGAACTCAGGCTGGGCATC
*IL-22*	TCCAACTTCCAGCAGCCATACATC	GGTAGCACTGATCCTTAGCACTGAC
*IL-17*	GACTCTCCACCGCAATGAAGAC	CTCTTCAGGACCAGGATCTCTTG
*IL-4*	ACCAGGAGCCATATCCACGGATG	GGTGTTCTTCGTTGCTGTGAGGAC
*IL-10*	CGGGAAGACAATAACTGCACCC	CGGTTAGCAGTATGTTGTCCAGC
*Β-actin*	ATCGCTGCGCTGGTCG	GTCCTTCTGACCCATTCCC

**Table 3:** Major peptides identified from hydrolysate have different molecular mass and amino acid combinations.

*Serial No.*	*Protein Accession Number*	*Peptides Detected in MS Spectra*	*Mol. Weight (Da)*	*Length*	*m/z*
*1*	QPB41107.1	R.IRELEDALDSERDGRVR.A	2028.035	17	508.0164
*2*	QPB41107.1	R. VEKEKQTLVVE.L	1300.724	11	434.5822
*3*	QPB41107.1	L.TQENFDLQHQVQ.E	1485.65	12	743.85
*4*	QPB41107.1	A.SNDDLKRQLDDETRQR.Q	1987.967	16	497.9995
*5*	QPB41107.1	A. LQADYDNLNVR.L	1319.647	11	660.8311
*6*	QPB41107.1	L.TQENFDLQHQVQ.E	1485.685	12	743.85
*7*	QPB41107.1	L.KMEEM (+15.99)EDLKR.R	1323.616	10	442.2125
*8*	QPB41107.1	A.LAQELEDARALLEGAER.A	1882.975	17	628.666
*9*	QPB41107.1	R. KQLEVEIR.E	1013.587	8	507.8008
*10*	QPB41107.1	R.VRDLEAELENEARRVR.E	1954.034	16	489.5162
*11*	ACT34361.1	K.Q(-17.03) LTEDHFLFNDSDRFLK.A	2107.001	17	703.342
*12*	ACT34361.1	Q. LTEDHFLFNDSDR.F	1607.722	13	804.8658
*13*	QPI34813.1	K. IDTFIDVQKDP.V	1289.65	11	645.8335

## Data Availability

The original data for this work is available upon email request to the corresponding author.
